# Advising vaccinations for the elderly: a cross-sectional survey on differences between general practitioners and physician assistants in Germany

**DOI:** 10.1186/s12875-016-0502-3

**Published:** 2016-07-29

**Authors:** Carolina Judith Klett-Tammen, Gérard Krause, Thomas von Lengerke, Stefanie Castell

**Affiliations:** 1 Department for Epidemiology, Helmholtz Centre for Infection Research, Inhoffenstr. 7, Braunschweig, 38124 Germany; 2 Institute for Epidemiology, Social Medicine and Health Systems Research, Hannover Medical School, Hannover, Germany; 3 Chair for Infectious Disease Epidemiology, Hannover Medical School, Hannover, Germany; 4 Medical Psychology Unit, Hannover Medical School, Carl-Neuberg-Str. 1, Hannover, 30625 Germany

**Keywords:** Vaccination, Elderly, Aged, General practitioners, Physician assistants, Health knowledge, Attitudes, Practice

## Abstract

**Background:**

In Germany, the coverage of officially recommended vaccinations for the elderly is below a desirable level. It is known that advice provided by General Practitioners and Physician Assistants influences the uptake in patients ≥60 years. Therefore, the predictors of advice-giving behavior by these professions should be investigated to develop recommendations for possible actions for improvement.

**Methods:**

We conducted a postal cross-sectional survey on knowledge, attitudes and advice - giving behavior regarding vaccinations in the elderly among General Practitioners and Physician Assistants in 4995 practices in Germany. To find specific predictors, we performed logistic regressions with non-advising on any officially recommended vaccination or on three specific vaccinations as four separate outcomes, first using all participants, then only General Practitioners and lastly only Physician Assistants as our study population.

**Results:**

Participants consisted of 774 General Practitioners and 563 Physician Assistants, of whom overall 21 % stated to have not advised an officially recommended vaccination in elderly patients. The most frequent explanation was having forgotten about it. The habit of not counselling on vaccinations at regular intervals was associated with not advising any vaccination (OR: 2.8), influenza vaccination (OR: 2.3), and pneumococcal vaccination (OR: 3.1). While more General Practitioners than Physician Assistants felt sufficiently informed (90 % vs. 79 %, *p* < 0.001), General Practitioners displayed higher odds to not advise specific vaccinations (ORs: 1.8–2.8).

**Conclusions:**

To reduce the high risk of forgetting to advice on vaccinations, we recommend improving and promoting standing recall-systems, encouraging General Practitioners and Physician Assistants to counsel routinely at regular intervals regarding vaccinations, and providing Physician Assistants with better, tailor-made information on official recommendations and their changes.

**Electronic supplementary material:**

The online version of this article (doi:10.1186/s12875-016-0502-3) contains supplementary material, which is available to authorized users.

## Background

Recommendations of the German Standing Committee on Vaccination (STIKO) include influenza vaccination (IV), tetanus vaccination (TV) and pneumococcal vaccination (PV) for individuals who are 60 years or older [1]. These officially recommended vaccinations are financially compensated by the statutory health insurances [2] as the benefit risk ratio of the recommended vaccines has been assessed to be positive [3, 4]. Although general practitioners (GP) are mostly self-employed and therefore have to care for the economic aspects of their practice, they receive an appointed amount for a specific service. As there are several associations of statutory health insurance physicians for different regions in Germany and each negotiates the compensation for the physicians for specific services with the statutory health insurances, the specific compensation for vaccinations might differ.

Vaccination coverage for PV in the elderly is as low as 31 % in Germany [5]; for IV, it is with 37 % clearly lower than the target of 75 % vaccination in the elderly [6], given by the World Health Organization [7]; while it is high for TV with 93 - 95 % [5]. Mostly, GP and physician assistants (PA) advice on vaccinations, and recommendations by these professions influence the vaccination uptake especially in the elderly [8–10]. In Germany, PA assist physicians regarding checkups, treatment, care and counselling of patients and organizational and administrational aspects, but do not treat or counsel autonomously. It can be assumed that in almost every non-private general practice in Germany, at least one PA works. PA themselves cannot open a practice and treat patients but only assist physicians [11]. However, while there is some basic evidence from the year 2000/2001 regarding knowledge, attitude, and practice (KAP) factors with respect to vaccinations in the elderly in German GP [12] and predictors for advising specific vaccines to individuals in this age-group in American [13] and Australian [14] physicians, PA have been neglected in vaccination-related research in Germany so far. To our knowledge, there has been no study analyzing vaccination-related KAP or KAP as predictors for advice-giving behavior towards the elderly in GP and PA for TV, IV and PV in a sample representative for Germany using multivariable analyses. Therefore, we conducted a survey in both professions in Germany and explored - within the KAP-framework - predictors for not giving vaccination advice to the elderly in general, and on TV, IV, and PV specifically, to gain insight into opportunities for profession specific improvement of advice-giving behavior.

## Methods

We developed two KAP-questionnaires (for GP and PA specifically), and piloted them, using cognitive pretest-interviews as think-aloud, comprehension, category selection and information retrieval probing and confidence rating [15] in 16 persons. We assembled a database comprising all German GP treating adult patients with statutory health insurance using publicly accessible data from the federal associations of statutory health insurance physicians and the medical councils; and then selected a random sample of 5000 practices, stratified and weighted for federal state. We mailed two questionnaires to each selected practice in March 2015. All questionnaire variables and their definitions are presented in Additional file 1.

We evaluated the representativeness of our study population by chi-square tests, using the atlas of physicians [16] for age-distribution and location of practice, i.e. working in East/West Germany with Berlin as East of GP, the statistics of physicians [17] for sex-distribution of the physicians, and the statistics of employees in health service [18] for sex- and age-distribution of PA as data on the source populations.

We performed logistic regressions for each of the four outcomes, i.e., reporting not having recommended any vaccination/TV/IV/PV despite official STIKO-recommendation and in the absence of contraindication in the elderly. Each regression was modelled three times: including all participants, only GP, and only PA, excluding PA that did report to be not responsible for vaccination counseling in their practice (*n* = 107). We included all variables with *p* < 0.25 in bivariate analyses (using chi^2^ -test for nominal variables, *t*-test for normally distributed metric, and Mann-Whitney-U for not normally distributed metric and ordinal variables, with the distribution tested graphically and by Shapiro-Wilk-test) and applied backward selection with *p* < 0.2 as model inclusion criteria. If more than 5 % of missing values in predictor variables occurred, we used chain-multiple imputations with five datasets for multivariable analyses. Otherwise and in bivariate analyses, we conducted complete case analyses. For multivariable analysis, we used the GP answer for the PA when GP and PA from the same practice stated to work in different parts of Germany (*n* = 272). We report associations with *p* < 0.05 as statistically significant. All analyses were carried out using Stata 12.

## Results

### Study population

**Fig. 1:**
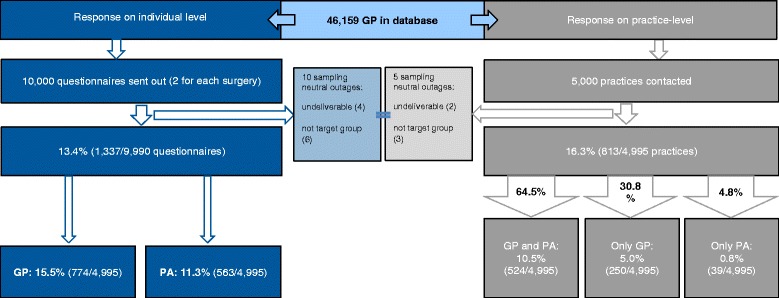
Description of response on individual and practice-level

Of the netto-sample of 4995 practices, 16.3 % returned questionnaires (813/4995) corresponding to 13.4 % eligible participants (1337/9990) (Fig. 1).

Median age of GP was 54 years (interquartile range (IQR): 48–61), median time working as GP was 17 years (IQR: 10–24), 47 % of the respondents were female and 77 % worked in the Western part of Germany. Median age of PA was 43 years (IQR: 30–51), median time working with a GP was 20 years (IQR: 10–30), and 97 % of the respondents were female.

Regarding age-distribution, we found no significant difference between our study population and the source population for both professions (all *p* > 0.05); regarding location of practice, we found no difference for GP (*p* > 0.05), while more female GP (47 % vs 43 %, *p* = 0.02) and more male PA (3 % vs. 2 %, *p* = 0.03) participated.

### Description of knowledge, attitude, and practices

Of all participants, 265 (22 %) stated to have not advised at least one vaccination to an elderly patient despite STIKO-recommendation and absence of a contraindication. PV was the vaccination most frequently not being suggested (*n* = 183), i.e. 15 % of all participants involved in counseling on vaccinations, or 19 % (148/774) of GP respectively. Most participants reported to know (92 %, 1235/1337) and to trust (90 %, 1200/1337) the STIKO-recommendations. Whereas 85 % (1140/1337) of the respondents felt in general sufficiently informed about vaccinations in the elderly, 66 % (880/1337) required better information on changes of STIKO-recommendations. Respondents supported (95 %, 1265/1337) discussions about vaccinations being initiated by patients and utilized this as an opportunity to counsel (92 %, 1232/1337) (Table 1).

**Table 1:** Knowledge, attitude, practice regarding vaccinations in the elderly in general practitioners (GP) and physician assistants (PA)

		Total, *n* = 1337	GP, *n* = 774	PA, *n* = 563	
		No. (%)	No. (%)	No. (%)	*p*-value*
Outcomes	Not advised at least one recommended vaccination in the elderly without presence of a contraindication^a^	265 (21.5)	201 (26.0)	64 (14.0)	**<0.001**
	*Missing*	37 (3.0)	16 (2.1)	21 (4.6)	
	Not advised tetanus vaccination in the elderly without presence of a contraindication^a^	90 (7.3)	76 (9.8)	14 (3.1)	**<0.001**
	Not advised influenza vaccination in the elderly without presence of a contraindication^a^	141 (11.5)	113 (14.6)	28 (6.1)	**<0.001**
	Not advised pneumococcal vaccination in the elderly without presence of a contraindication^a^	183 (14.9)	148 (19.1)	35 (7.7)	**<0.001**
KL	Knows official STIKO-recommendations	1235 (92.4)	752 (97.2)	483 (85.8)	**<0.001**
	*Missing*	33 (2.5)	17 (2.2)	16 (2.8)	
	Feels sufficiently informed regarding vaccinations in adults	1140 (85.3)	696 (89.9)	444 (78.9)	**<0.001**
	*Missing*	34 (2.5)	19 (2.5)	15 (2.7)	
Attitudes	Patients should be informed by (multiple answers possible)				
	*GP*	1308 (97.8)	755 (97.6)	553 (98.2)	0.400
	*Other physician or specialist*	724 (54.2)	451 (58.3)	273 (48.5)	**<0.001**
	*PA*	921 (68.9)	493 (63.7)	428 (76.0)	**<0.001**
	*Health insurance company*	873 (65.3)	503 (65.0)	370 (65.7)	0.781
	Appreciates it if patients address vaccinations.	1265 (94.6)	746 (96.4)	519 (92.2)	**<0.001**
	*Missing*	40 (3.0)	21 (2.7)	19 (3.4)	
	Trusts official STIKO-recommendations.	1200 (89.8)	706 (91.2)	494 (87.7)	**0.005**
	*Missing*	33 (2.5)	20 (2.6)	13 (2.3)	
	Wants more information for patients by public authorities.	686 (51.3)	369 (47.7)	317 (56.3)	**0.001**
	*Missing*	61 (4.6)	35 (4.5)	26 (4.6)	
	Finds financial compensation for advising and vaccinating sufficient.	195 (14.6)	122 (15.8)	73 (13.0)	**<0.001**
	*Missing*	90 (6.7)	33 (4.3)	57 (10.1)	
	Wants better information on changes of official recommendations.	880 (65.8)	503 (65.0)	377 (67.0)	0.240
	*Missing*	47 (3.5)	25 (3.2)	22 (3.9)	
	There is often lack of time for vaccinations and advising about them.	373 (27.9)	178 (23.0)	195 (34.6)	**<0.001**
	*Missing*	33 (2.5)	22 (2.8)	11 (2.0)	
	General objection of vaccinations	13 (1.0)	5 (0.6)	8 (1.4)	**0.038**
	*Missing*	82 (6.1)	44 (5.7)	38 (6.7)	
	Perceived benefit of all officially recommended vaccines exceeds its potential harms	1034 (77.3)	666 (86.0)	368 (65.4)	**<0.001**
	*Missing*	52 (3.9)	29 (3.7)	23 (4.1)	
	Perceived benefit of officially recommended influenza-vaccine exceeds its potential harms	1064 (79.6)	640 (82.7)	424 (75.3)	**<0.001**
	*Missing*	43 (3.2)	31 (4.0)	12 (2.1)	
	Perceived benefit of officially recommended pneumococcal-vaccine exceeds its potential harms	1051 (78.6)	643 (83.1)	408 (72.5)	**<0.001**
	*Missing*	47 (3.5)	26 (3.4)	21 (3.7)	
	Perceived benefit of officially recommended tetanus-vaccine exceeds its potential harms	1156 (86.5)	724 (93.5)	432 (76.7)	**<0.001**
	*Missing*	19 (1.4)	0	19 (3.4)	
	Cause that vaccination recommendations are not well implemented				**<0.001**
	*Physicians*	74 (5.5)	51 (6.6)	23 (4.1)	
	*Patients*	376 (28.1)	162 (20.9)	214 (38.0)	
	*Both*	757 (56.6)	510 (65.9)	247 (43.9)	
	*None of them*	89 (6.7)	31 (4.0)	58 (10.3)	
	*Missing*	41 (3.1)	20 (2.6)	21 (3.7)	
	Likes to counsel about vaccinations^a^	1078 (87.6)	683 (88.2)	395 (86.6)	0.299
	*Missing*	27 (2.2)	17 (2.2)	10 (2.2)	
Practices	Opportunities for vaccinations counselling (multiple answers possible)				
	*Patient addresses it*	1232 (92.1)	710 (91.7)	522 (92.7)	0.508
	*Travel plans*	1225 (91.6)	701 (90.6)	524 (93.1)	0.103
	*Preventive checkup*	1205 (90.1)	715 (92.4)	490 (87.0)	**0.001**
	*Injuries*	1198 (89.6)	687 (88.8)	511 (90.8)	0.236
	*First contact with patient*	716 (53.16)	442 (57.1)	274 (48.7)	**0.002**
	*Indication of a recall-system*	312 (23.3)	170 (22.0)	142 (25.2)	0.164
	*Routinely at regular intervals*	225 (16.8)	124 (16.0)	101 (17.9)	0.354
	Source of information regarding vaccines (multiple answers possible)				
	*Continuous (online) training*	756 (56.5)	464 (59.9)	292 (51.9)	**0.003**
	*Professional journals*	812 (60.7)	578 (74.7)	234 (41.6)	**<0.001**
	*Conferences*	354 (26.5)	305 (39.4)	49 (8.7)	**<0.001**
	*Pharmaceutical representative*	692 (51.8)	359 (46.4)	333 (59.1)	**<0.001**
	*Professional association*	147 (11.0)	107 (13.8)	40 (7.1)	**<0.001**
	*STIKO*	1050 (78.5)	662 (85.5)	388 (68.9)	**<0.001**

*GP* General Practitioner, *PA* Physician Assistant, *KL* Knowledge

*p-values < 0.05 in bold letters (Chi^2^-Test for differences between GP and PA without missings)

^a^PA excluded that not reported to be (partly) responsible for vaccination advices in their practice (*n* = 107)

The most common explanations given by respondents for not advising was for all three investigated vaccinations forgetting to advise (53–72 %), followed by the perceived low risk of the patient to catch the respective disease (23–28 %). Uniquely for IV, 19 participants (14 %) stated to not have advised it due to safety concerns and 21 (15 %) due to doubts on its effectiveness (Table 2).

**Table 2:** Reasons for not advising specific vaccinations despite STIKO-recommendation and in the absence of any contraindication (multiple answers possible)

	Total^a^ (*n* = 1230)			General practitioners (*n* = 774)			Physician assistants^a^ (*n* = 456)		
	Tetanus (*n* = 90)	Influenza (*n* = 141)	Pneumococcal (*n* = 183)	Tetanus (i = 76)	Influenza (*n* = 113)	Pneumococcal (*n* = 148)	Tetanus (*n* = 14)	Influenza (*n* = 28)	Pneumococcal (*n* = 35)
	No. (%)	No. (%)	No. (%)	No. (%)	No (%)	No (%)	No. (%)	No (%)	No. (%)
Disease is harmless	0	4 (2.8)	3 (1.6)	0	2 (1.8)	2 (1.4)	0	2 (7.1)	1 (2.9)
Forgot to advise	65 (72.2)	74 (52.5)	129 (70.5)	56 (73.7)	60 (53.1)	107 (72.3)	9 (64.3)	14 (50.0)	22 (62.9)
Vaccine is ineffective	2 (2.2)	21 (14.9)	9 (4.9)	2 (2.6)	19 (16.8)	9 (6.1)	0	2 (7.1)	0
Does not feel responsible for vaccinations	7 (7.8)	3 (2.1)	4 (2.2)	5 (6.6)	0	0	2 (14.3)	3 (10.7)	4 (11.4)
Vaccine is not safe	1 (1.1)	19 (13.5)	12 (6.6)	1 (1.3)	13 (11.5)	10 (6.8)	0	6 (21.4)	2 (5.7)
Was not aware of recommendation	5 (5.6)	2 (1.4)	4 (2.2)	5 (6.6)	2 (1.8)	2 (1.4)	0	0	2 (5.7)
Risk of the patient to catch disease is low	21 (23.3)	38 (27.0)	51 (27.9)	18 (23.7)	33 (29.2)	43 (29.1)	3 (21.4)	5 (17.9)	8 (22.9)
Accounting is too complicated	3 (3.3)	3 (2.1)	3 (1.6)	1 (1.3)	1 (0.9)	1 (0.7)	2 (14.3)	2 (7.1)	2 (5.7)
Reimbursement does not compensate the effort	8 (8.9)	11 (7.8)	9 (4.9)	4 (5.3)	7 (6.2)	4 (2.7)	4 (28.6)	4 (14.3)	5 (14.3)

^a^Only Physician Assistants included that stated to be (partly) responsible for vaccination advises in their practice

### Predictors for not advising vaccinations

In multivariable analyses, odds ratios (OR) >1 imply that the influencing factor increases the chance of not having advised a vaccination to elderly, while OR <1 signify an increasing chance that a vaccination is always advised when medically indicated and recommended by STIKO.

**Fig. 2:**
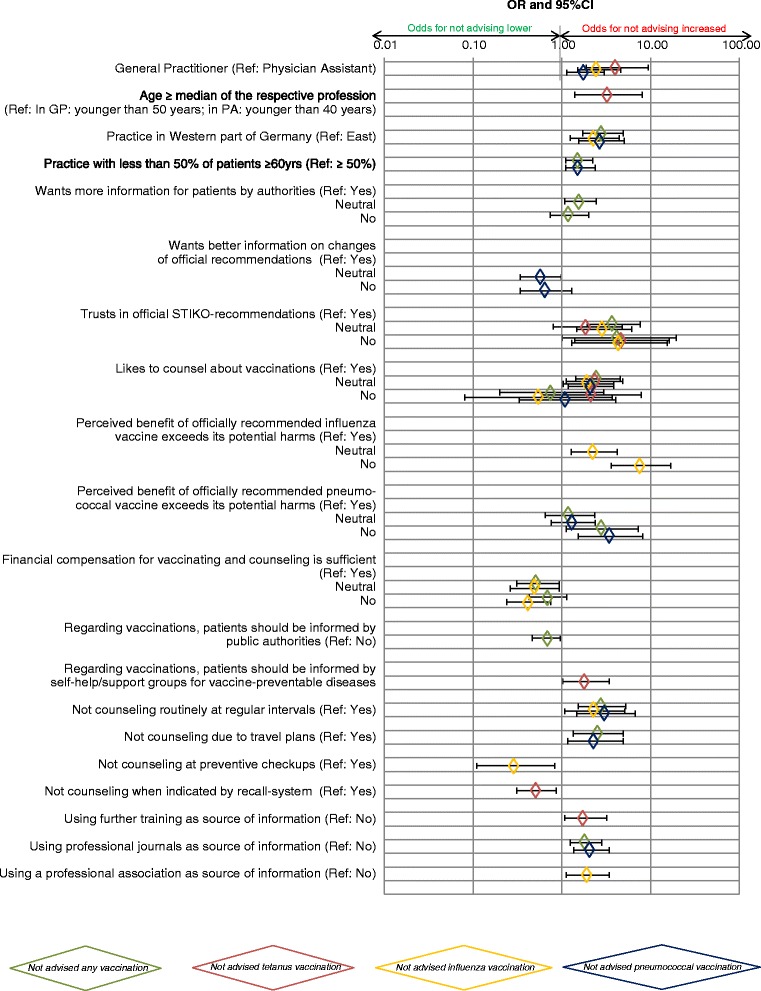
Results from multivariable models. Legend: Diamonds signify the four different outcomes, i.e. vaccinations in the elderly despite STIKO-recommendation and in the absence of contraindications. OR and 95 % CI are displayed. Non-significant results (*p* ≥ 0.05) are added if any level of a variable yielded a significant result. Logarithmic scale

Most prominent predictor for any (4.4, 1.0–19.4) and tetanus vaccination (4.9, 1.5–16.5) is not trusting the STIKO-recommendations; although this concerns only 2 % of respondents (25/1337). For influenza (7.8, 3.6–16.9) and pneumococcal vaccination (3.5, 1.5–8.2), the negative perceived benefit-harm-ratio of the respective vaccine showed the most substantial association for not advising it to the elderly. While working in West-Germany more than doubles the odds for not advising any vaccination (2.9, 1.7–4.9), IV (2.4, 1.3–4.5) and PV (2.8, 1.6–5.1), this association is not significant in the model for TV. Not counseling on vaccinations at regular intervals, e.g. at the first visit of a patient within an accounting period, is associated with not advising any vaccination (2.8, 1.5–5.3), IV (2.3, 1.1–5.1), and PV (3.1, 1.5–6.7). GP exhibit about two times the odds for not having advised specific vaccinations compared to PA (TV: 2.8, 1.5–5.4; IV: 2.6, 1.5–4.6; PV: 1.8, 1.1-3.0) (Fig. 2, Additional file 2).

### Comparison of general practitioners and physician assistants

PA supported GP regarding counseling on vaccinations in 79 % (612/813) of responding practices. Despite the higher chance of GP to not have advised all investigated vaccinations in the elderly compared to PA, more GP than PA felt sufficiently informed regarding vaccinations in adults (90 % of GP (696/774) vs. 79 % of PA (444/563), *p* < 0.001), stated to know STIKO-recommendations (97 % of GP (752/774) vs. 86 % of PA (483/563), *p* < 0.001), and to use them as a source of information (85 % of GP (662/774) vs. 69 % of PA (388/563), *p* < 0.001). For all investigated vaccinations, less PA than GP believed the benefit to exceed potential harms (all *p* < 0.001) (Table 1).

Modelling GP and PA separately indicates that e.g. location of the practice in the Western part of Germany, and not counseling routinely at regular intervals increases the chance for not advising on any vaccination only in GP, whereas e.g. age-structure of practices’ patients is only a significant predictor in PA (Table 3, Additional file 3).

**Table 3:** Multivariable models for each, GP and PA

		GP (*n* = 774)		PA that are responsible for vaccination advices in their surgery (*n* = 456)	
	Variables	OR (95 % CI)	*p*-value	OR (95 % CI)	*p*-value
SD	Practice in Western part of Germany (Ref: East)	2.81 (1.59–4.97)	<0.001	n.s.	
	Amount of patients of ≥60 year less than 50 % (Ref: ≥50 %)	n.s.		2.19 (1.20–4.00)	0.011
Attitudes	Trusts in official recommendations			n.s.	
	Ref: Yes	1 (Ref)			
	Neutral	3.61 (1.56–8.32)	0.003		
	No	19.30 (2.28–163.24)	0.007		
	Likes to counsel about vaccinations			n.s.	
	Ref: Yes	1 (Ref)			
	Neutral	2.94 (1.56–5.54)	0.001		
	*No*	*1.02 (0.24–4.27)*	*0.976*		
	Benefit of officially recommended pneumococcal vaccine exceeds its potential harms			n.s.	
	Ref: Yes	1 (Ref)			
	*Neutral*	*1.85 (0.99–3.44)*	*0.054*		
	No	3.26 (1.30–8.14)	0.012		
	Regarding vaccinations, patients should be informed by:				
	General Practitioner (Ref: No)	0.31 (0.10–0.94)	0.038	n.s.	
	Public authorities (Ref: No)	n.s.	.	0.43 (0.21–0.90)	0.026
P	Not counseling routinely at regular intervals (Ref: Yes)	2.86 (1.37–5.96)			

Statistically significant (defined as *p* < 0.05) associations with not advising at least one vaccination in the elderly despite STIKO-recommendations and no contraindication modelled separately for General Practitioner (GP) and Physician Assistant (PA); non-significant results (*p* ≥ 0.05) are added if any level of a variable yielded a significant result and are shown in italics

*Ref* Reference, *SD* Socio-demographic and practice-characteristics, *P* Practice, *n.s.* not significant

## Discussion

Over 20 % of the participants stated that they had not advised at least one officially recommended vaccination, even in absence of any specific contraindication; with 26 % of the GP and 14 % of the PA, significantly more physicians than assistants reported a vaccination-advise practice deviating from recommendations. By far the most frequent explanation in both professions was “having forgotten to advise”. More than 20 % of PA did not feel sufficiently informed regarding vaccinations in adults (vs. 10 % of the GP); 90 % of the respondents stated that they trusted the official STIKO-recommendations, and very few were general opponents of vaccination. Only 17 % of the participants counsel routinely at regular intervals and just 23 % use a recall-system. In general, the chance of not advising is higher in practices in West-Germany and with younger patients. Also, those who do not counsel routinely at regular intervals, those with a neutral attitude towards counseling, and who do not trust the STIKO-recommendations have a higher chance of not advising. Overall, associations with KAP-variables were rather similar across different vaccines, while we observed some significant distinctions between GP and PA. The high proportion of GP and PA working in the Western part of Germany can be explained by the general high proportion of the German population living there (~16 million in the Eastern part versus ~65 million people in the Western part [19]).

There are only few vaccination-related KAP-surveys among GP that address vaccinations in the elderly. A German study, published 16 years ago, found the same geographic difference in following the official recommendations as we did, with GP in the Western part of Germany vaccinating/advising less often than in the east [12]. This matches the observation of general higher vaccination coverage in the Eastern part of Germany [5]. To the 18 % of their respondents never vaccinating against pneumococci, the 19 % of GP in our study, who reported that they had not advised PV (mostly due to having forgotten to do so), seem to be comparable, although the outcomes are not exactly the same, as we did not ask about the actual vaccination, but about advising vaccinations. Opportunities for vaccination were similar to our study, e.g. a majority of 84 % stated to counsel during preventive check-ups and 71 % at first contact with a patient, although, with only 4 %, even less participants stated to counsel routinely at regular intervals [12]. The association of the perceived benefit-harm-ratio of the corresponding vaccine with not advising IV and PV which we saw in our results was matched by the result of a survey in the USA [13], where mainly belief-related predictors as perceived vaccine-effectiveness for advising IV and PV were found. In Australia, most common explanations for not giving vaccinations to the elderly by GPs were refusal of patients (88 %) and competing priorities (35 %) [14]. In a Canadian study, addressing mainly childhood vaccinations, nurses were also included, showing a more positive attitude towards administering different vaccinations during a single visit than physicians [20], which is in line with the result in our study that less PA than physicians reported advice-failures. As the PA in our study did not show a substantially more positive attitude towards vaccinations, the lower proportion of reported non-advising than in GP could demonstrate a more rule-governed behavior of the PA, a higher vulnerability to a social desirability bias or less knowledge of norms and therefore less conscious deviation of these norms.

So far, PA have been neglected in public health vaccinology in Germany; however, as they also counsel and administer vaccinations [21, 22], it would be beneficial to include them in future activities or interventions regarding vaccine-uptake. Our study has generated new knowledge concerning the needs for information of PA and points out attitudes that can be useful to optimize future interventions. Since our study included physicians and their assistants, using mostly the same questions, we were able to compare both professions systematically with respect to knowledge, attitudes and practices. Thus, we found statistically significant differences not only in advice-giving behavior, but also in subjective knowledge of official recommendations and the trust they are met with. This also applies to other aspects like the perceived benefit-harm-ratio of certain vaccines, and practices like the sources of information regarding vaccinations.

### Strengths and limitations

The evaluation of the representativeness regarding age, sex and location of practice by chi-square tests did not indicate any bias in recruitment or response within the German population of GP and PA, despite the low response with 11.3 % in PA and 15.5 % in GP. Still, the variables available for investigating representativeness do not necessarily represent characteristics relevant for the research question at hand. Furthermore, using other statistical test methods might result in different findings. The generalizability of our results is therefore limited.

Due to known difficulties in recruiting GPs for such studies in Germany [23], we chose not to test the knowledge regarding vaccinations, in order not to embarrass and thus repel possible participants. However, we assume our design, i.e. asking how well subjects feel informed, to describe this factor sufficiently well. As we merely assessed self-reported advice-giving behavior, not actually observing the routine, unintentional misperceptions by the participants regarding recommendations, contraindications, or their own behavior, biased responses might be possible. To avoid bias due to lack of awareness of the recommendations for TV, IV and PV, we specifically asked if a vaccination had ever *not* been recommended to a person of at least 60 years, despite the absence of a contraindication. We also provided the option to state that the participant did not know about above-mentioned recommendations. Still, due to social desirability or recall problems an underestimation of not advising vaccinations by GP and PA is possible.

## Conclusions

By far the most frequent explanation given for not advising a vaccination was forgetting about it, matching the substantial association of not advising any, influenza and pneumococcal vaccination with not counseling routinely at regular intervals. Still, despite improvements in this field (compared to 2000 [12]), only a minority of participants stated that they counselled regularly or used a recall-system. As it is known that many opportunities for counseling on vaccinations are not used [24], an easy-to-implement automated recall-feature complying with legal requirements and integrated in the practice management system seems to be an absolute necessity of modern health care administration. Furthermore, it has to be promoted that these functions exist and how and under which circumstances they can be used for reminding patients and alerting GP and PA [25, 26] and if there are new developments as automated management of appointments including possibilities for recalls [27]. In most practices, PA also counsel on vaccination, but seem to feel insufficiently informed about vaccinations in adults or changes in STIKO recommendations. Therefore the provision of better edited information (e.g. on efficacy and safety), tailored specifically to the needs of PA might improve the situation significantly. Another option to improve the vaccination rates in the elderly could be to allow for other health care specialists to apply specific vaccinations, as public health services. In Ireland, pharmacists are involved [28] with good results for influenza vaccination [29, 30]. However, in some areas physicians seem to be the most important source of vaccinations and vaccination counselling [31].

Empowering PA, installing and promoting mechanisms to reduce the risk of forgetting to give vaccination advice and include special vaccination hours in public health services in the vaccinations procedure may open up new avenues to improved vaccination coverage in the elderly.

## Abbreviations

GP, general practitioner; IV, influenza vaccination; P, practice; PA, physician assistant; PV, pneumococcal vaccination; SD, socio-demographic and practice-characteristics; STIKO, German standing committee on vaccination; TV, tetanus vaccination.

## Additional files

Additional file 1: Variables: definitions and origins. Description: List of all variables, their definitions and origins. (PDF 239 kb) Additional file 2: Multivariable analyses of associations with not advising vaccinations despite STIKO-recommendation. Description: Full models including non-significant associations with not advising specific vaccinations. *N* = 1337. (PDF 466 kb) Additional file 3: Multivariable analyses of associations with not advising vaccinations despite STIKO-recommendation in GPs and PAs separately. Description: Full models including non-significant associations with not advising specific vaccinations. (PDF 446 kb)
