# Supplementary material for: Advising vaccinations for the elderly: a cross-sectional survey on differences between general practitioners and physician assistants in Germany
Source: BMC Fam Pract. 2016 Jul 29;17:98. doi: 10.1186/s12875-016-0502-3 (PMC4966563; doi:10.1186/s12875-016-0502-3)
Supplement: Additional file 1: — Variables: definitions and origins. Description: List of all variables, their definitions and origins. (PDF 239 kb) [file 12875_2016_502_MOESM1_ESM.pdf]

Additional File 1: Variables: definitions and origins

Variable	Scale/Categories	Comment/Explanation
**Socio-demographic and surgery-characteristics**		
ID	-	Practice ID
Profession	• General Practitioner<br>• Physician Assistant	-
Counseling is done by	• Physician<br>• Physician Assistant<br>• Both<br>• Other Person	-
Percentage of patients of at least 60 years	metric	Estimate of participant
Amount of patients of at least 60 years	• < 50%<br>• ≥50%	dichotomized; derived from variable "Percentage of patients of at least 60 years"
Years working in outpatient care	metric	derived from year in which participant started working in outpatient care
Age	metric	-
Derived variable: Age	• < median of profession<br>• ≥median of profession	dichotomization in each profession by rounded median<br>General Practitioner: 50 years<br>Physician Assistant: 40 years
Specialty	• General Medicine<br>• Internist<br>• No specialization<br>• Other	In questionnaire for Physician Assistants we asked for the specialty of the respective Physician
Location of Practice	• Eastern part of Germany (including Berlin)<br>• Western part of Germany	Derived from reported federal state
**Subjective Knowledge**		
Knows official STIKO-recommendations	• Yes<br>• Neutral<br>• No	Aggregated Likert-Scale:<br>Yes: Applies<br>Rather applies<br>Neutral<br>No: Does rather not apply<br>Does Not apply
Feels sufficiently informed regarding vaccinations for adults	• Yes<br>• Neutral<br>• No	Aggregated Likert-Scale:<br>Yes: Applies<br>Rather applies<br>Neutral<br>No: Does rather not apply<br>Does Not apply
**Attitudes**		
Patients should be informed by	• General Practitioner<br>• Other medical specialist	Multiple answers possible<br>Public Authorities: examples in

	• Public Authorities<br>• Physician Assistant<br>• Self-help/support groups for vaccine-preventable diseases<br>• Media<br>• Health insurance companies<br>• Other	questionnaire: Robert-Koch institute and Federal Centre for Health Education
Appreciates it if patients start the conversation about vaccinations	• Yes<br>• Neutral<br>• No	Aggregated Likert-Scale:<br>Yes: Applies<br>Rather applies<br>Neutral<br>No: Does rather not apply<br>Does Not apply
Trusts official STIKO-recommendations	• Yes<br>• Neutral<br>• No	Aggregated Likert-Scale:<br>Yes: Applies<br>Rather applies<br>Neutral<br>No: Does rather not apply<br>Does Not apply
Wants more patient information by public authorities	• Yes<br>• Neutral<br>• No	Aggregated Likert-Scale:<br>Yes: Applies<br>Rather applies<br>Neutral<br>No: Does rather not apply<br>Does Not apply
Finds financial compensation for advising and vaccinating sufficient	• Yes<br>• Neutral<br>• No	Aggregated Likert-Scale:<br>Yes: Applies<br>Rather applies<br>Neutral<br>No: Does rather not apply<br>Does Not apply
Likes to counsel on vaccinations	• Yes<br>• Neutral<br>• No	Aggregated Likert-Scale:<br>Yes: Applies<br>Rather applies<br>Neutral<br>No: Does rather not apply<br>Does Not apply
Thinks that the additional training for Physician assistants to become an assistant for vaccinations is useful	• Yes<br>• Neutral<br>• No	Aggregated Likert-Scale:<br>Yes: Applies<br>Rather applies<br>Neutral<br>No: Does rather not apply<br>Does Not apply
Wants better information on changes of official vaccination recommendations	• Yes<br>• Neutral<br>• No	Aggregated Likert-Scale:<br>Yes: Applies<br>Rather applies

		Neutral<br>No: Does rather not apply<br>Does Not apply
There is often lack of time for vaccinations and advising about them	• Yes<br>• Neutral<br>• No	Aggregated Likert-Scale:<br>Yes: Applies<br>Rather applies<br>Neutral<br>No: Does rather not apply<br>Does Not apply
Generally objects vaccinations	• Yes<br>• Neutral<br>• No	Aggregated Likert-Scale:<br>Yes: Applies<br>Rather applies<br>Neutral<br>No: Does rather not apply<br>Does Not apply
The perceived benefit of officially recommended vaccines exceeds its potential harms	• Yes<br>• Neutral<br>• No	Aggregated Likert-Scale:<br>Yes: Applies<br>Rather applies<br>Neutral<br>No: Does rather not apply<br>Does Not apply
The perceived benefit of officially recommended tetanus vaccine for at least 60-years old individuals exceeds its potential harms	• Yes<br>• Neutral<br>• No	Aggregated Likert-Scale:<br>Yes: Applies<br>Rather applies<br>Neutral<br>No: Does rather not apply<br>Does Not apply
The perceived benefit of officially recommended influenza vaccines for at least 60-years old individuals exceeds its potential harms	• Yes<br>• Neutral<br>• No	Aggregated Likert-Scale:<br>Yes: Applies<br>Rather applies<br>Neutral<br>No: Does rather not apply<br>Does Not apply
The perceived benefit of officially recommended pneumococcal vaccines for at least 60-years old individuals exceeds its potential harms	• Yes<br>• Neutral<br>• No	Aggregated Likert-Scale:<br>Yes: Applies<br>Rather applies<br>Neutral<br>No: Does rather not apply<br>Does Not apply
Cause that vaccination recommendations are not well implemented	• Physicians<br>• Patients<br>• Both<br>• None of them	
Did ever not advise tetanus vaccination because	• Disease is harmless<br>• Forgot to advise<br>• Vaccine is ineffective<br>• Does not feel responsible for this vaccinations	Only applied when participant stated to have not advised at least one vaccination (see below). Multiple answers possible.

	• Vaccine is not safe<br>• Was not aware of recommendation<br>• Risk of the patient to catch disease is low<br>• Accounting is too complicated<br>• Reimbursement does not compensate the effort	
Did ever not advise influenza vaccination because	• Disease is harmless<br>• Forgot to advise<br>• Vaccine is ineffective<br>• Does not feel responsible for vaccinations<br>• Vaccine is not safe<br>• Was not aware of recommendation<br>• Risk of the patient to catch disease is low<br>• Accounting is too complicated<br>• Reimbursement does not compensate the effort	Only when participant stated to have not advised at least one vaccination. Multiple answers possible, ticked when agreed, every reason coded as one variable
Did ever not advise Pneumococcal vaccination because	• Disease is harmless<br>• Forgot to advise<br>• Vaccine is ineffective<br>• Does not feel responsible for vaccinations<br>• Vaccine is not safe<br>• Was not aware of recommendation<br>• Risk of the patient to catch disease is low<br>• Accounting is too complicated<br>• Reimbursement does not compensate the effort	Only when participant stated to have not advised at least one vaccination. Multiple answers possible, ticked when agreed, every reason coded as one variable
**Practices**		
Did not advise at least one vaccination in the absence of any contraindication.	• Yes<br>• No	Did not advise “once”and ”several times” were aggregated. In the question it was specified, that officially recommended vaccinations for the elderly (≥60 years) are of interest, if no contraindication was present.
Did not advise Tetanus vaccination in the absence of any contraindication.	• Yes<br>• No	Derived from “Did ever not advise Tetanus vaccination because”. If a reason was ticked,

		this variable was coded as "yes"
Did not advise Influenza vaccination in the absence of any contraindication.	• Yes<br>• No	Derived from "Did ever not advise Influenza vaccination because". If a reason was ticked, this variable was coded as "yes"
Did not advise Pneumococcal vaccination in the absence of any contraindication.	• Yes<br>• No	Derived from "Did ever not advise Pneumococcal vaccination because". If a reason was ticked, this variable was coded as "yes"
Opportunities for vaccination counseling	• Preventive Check-up<br>• Patient addresses vaccination<br>• Travel plans of patients<br>• First contact with a patient<br>• Injuries/accidents<br>• Routinely at regular intervals<br>• Indication of a recall-system<br>• Other	Multiple answers possible
Source of information regarding recommended vaccines	• Continuous (online) training<br>• Professional journals<br>• Association of Statutory Health Insurance Physicians or Medical Association<br>• Conferences<br>• Pharmaceutical /company representatives<br>• Professional association<br>• Permanent vaccination commission/Robert Koch-Insitute<br>• Does not seek information at all<br>• Other	Multiple answers possible
