# Supplementary material for: Advising vaccinations for the elderly: a cross-sectional survey on differences between general practitioners and physician assistants in Germany
Source: BMC Fam Pract. 2016 Jul 29;17:98. doi: 10.1186/s12875-016-0502-3 (PMC4966563; doi:10.1186/s12875-016-0502-3)
Supplement: Additional file 2: — Multivariable analyses of associations with not advising vaccinations despite STIKO-recommendation. Description: Full models including non-significant associations with not advising specific vaccinations. N = 1337. (PDF 466 kb) [file 12875_2016_502_MOESM2_ESM.pdf]

Additional File 2: Multivariable analyses of associations with not advising vaccinations despite STIKO-recommendation, n=1230

		**Did not advise any vaccination**		**Did not advise tetanus vaccination**		**Did not advise influenza vaccination**		**Did not advise pneumococcal vaccination**	
	**Variables**	**OR (95%CI)**	**p-value**	**OR (95%CI)**	**p-value**	**OR (95%CI)**	**p-value**	**OR (95%CI)**	**p-value**
**SD**	Respondent is GP (Reference: PA)	1.43 (0.91-2.22)	0.118	2.81 (1.46-5.42)	**0.002**	2.63 (1.51-4.58)	**0.001**	1.84 (1.13-3.01)	**0.015**
	Practice in Western part of Germany (Reference: East)	2.91 (1.72-4.92)	**<0.001**	1.77 (0.88-3.57)	0.110	2.36 (1.25-4.45)	**0.008**	2.80 (1.55-5.06)	**0.001**
	Amount of patients of ≥60 yrs less than 50% (Reference: ≥50%)	1.57 (1.10-2.23)	**0.013**					1.61 (1.10-2.37)	**0.015**
	Age at least at the median of the respected profession (Reference: In GP: younger than 50 years; in PA: younger than 40 years)			1.75 (1.00-3.06)	**0.049**				
**Attitudes**	Wants more information for patients by public authorities								
	Reference: Yes								
	Neutral	1.62 (1.08-2.42)	**0.019**					1.52 (0.98-2.35)	0.064
	No	1.22 (0.74-1.99)	0.435					1.31 (0.77-2.22)	0.321
	Wants better information on changes of official recommendations								
	Reference: Yes								
	Neutral	0.62 (0.39-1.01)	0.055	0.53 (0.26-1.04)	0.065			0.58 (0.34-0.98)	**0.041**
	No	0.77 (0.43-1.36)	0.363	0.70 (0.30-1.61)	0.400			0.67 (0.34-1.30)	0.234
	Trusts in official STIKO-recommendations								
	Reference: Yes								
	Neutral	3.86 (1.94-7.65)	**<0.001**	2.00 (0.83-4.85)	0.124	2.98 (1.46-6.08)	**0.003**	1.55 (0.73-3.27)	0.251
	No	4.43 (1.01-19.42)	**0.048**	4.89 (1.45-16.49)	**0.010**	4.45 (1.29-15.39)	**0.018**	2.86 (0.78-10.49)	0.113
	Likes to counsel about vaccinations								
	Reference: Yes								
	Neutral	2.56 (1.44-4.54)	**0.001**	2.09 (1.01-4.34)	**0.047**	1.98 (1.03-3.82)	**0.041**	2.13 (1.18-3.84)	**0.012**
	No	0.77 (0.20-2.97)	0.699	2.20 (0.62-7.81)	0.223	0.55 (0.08-3.70)	0.538	1.16 (0.33-4.06)	0.818
	Perceived benefit of officially recommended influenza vaccine exceeds its potential harms								
	(*Reference: Yes)*								
	Neutral	0.96 (0.50-1.86)	0.906			2.33 (1.28)	**0.005**		
	No	2.00 (0.76-5.25)	0.159			7.79 (3.59-16.93)	**<0.001**		
	Benefit of officially recommended pneumococcal vaccine exceeds its potential harms								
	Reference: Yes								
	Neutral	1.24 (0.65-2.35)	0.511					1.34 (0.76-2.37)	0.314
	No	2.84 (1.11-7.28)	**0.030**					3.54 (1.52-8.20)	**0.003**
	Regarding vaccinations, patients should be informed by:								
	Other medical specialist			1.43 (0.85-2.40)	0.176				
	Health insurance company							0.74 (0.49-1.10)	0.131
	Groups of self-help for vaccine-preventable diseases			1.85 (1.02-3.37)	**0.043**				
	Public authorities	0.67 (0.46-0.96)	**0.031**						
	Financial compensation for vaccinating and counseling is sufficient								
	Reference: Yes								
	Neutral	0.53 (0.31-0.93)	0.027			0.49 (0.26-0.93)	**0.029**		
	No	0.69 (0.43-1.13)	0.142			0.42 (0.24-0.75)	**0.003**		
**Practices**	Not counseling routinely at regular intervals	2.83 (1.52-5.26)	**0.001**	1.97 (0.87-4.46)	0.106	2.34 (1.08-5.10)	**0.032**	3.14 (1.46-6.74)	**0.003**
	Not counseling due to travel plans	2.56 (1.34-4.91)	**0.005**					2.39 (1.16-4.91)	**0.018**
	Not counseling when indicated by recall-system			0.50 (0.30-0.82)	**0.007**				
	Not counseling at preventive checkups	0.52 (0.25-1.10)	0.087			0.30 (0.11-0.83)	**0.021**	0.54 (0.25-1.19)	0.126
	Not counseling at first visit of patient	0.75 (0.52-1.07)	0.112						
	Not counseling when patient addresses it							0.41 (0.15-1.12)	0.084
	Source of information regarding vaccines								
	Continuing training			1.78 (1.04-3.05)	**0.034**				
	Pharmaceutical representative	0.75 (0.52-1.07)	0.108						
	Professional journals	1.83 (1.24-2.81)	**0.003**	1.64 (0.90-2.98)	0.109	1.63 (1.0-2.69)	0.055	2.16 (1.36-3.43)	**0.001**
	Professional association					1.95 (1.11-3.43)	**0.021**	1.58 (0.95-2.63)	0.077
	Specialty: No specialization			0.17 (0.02-1.31)	0.088				

Statistical significant Odds Ratios (p<0.05) are shown in bold. SD=Sociodemographic and practice-characteristics
