# Supplementary material for: Advising vaccinations for the elderly: a cross-sectional survey on differences between general practitioners and physician assistants in Germany
Source: BMC Fam Pract. 2016 Jul 29;17:98. doi: 10.1186/s12875-016-0502-3 (PMC4966563; doi:10.1186/s12875-016-0502-3)
Supplement: Additional file 3: — Multivariable analyses of associations with not advising vaccinations despite STIKO-recommendation in GPs and PAs separately. Description: Full models including non-significant associations with not advising specific vaccinations. (PDF 446 kb) [file 12875_2016_502_MOESM3_ESM.pdf]

Additional File 3: Multivariable analyses of associations with not advising vaccinations despite STIKO-recommendation in GPs and PAs separately.

		GP (n=774)								PA that are (partly) responsible for vaccination advices in their surgery (n= 456)							
		Did not advise any vaccination		Did not advise Tetanus vaccination		Did not advise Influenza vaccination		Did not advise pneumococcal vaccination		Did not advise any vaccination		Did not advise Tetanus vaccination		Did not advise Influenza vaccination		Did not advise pneumococcal vaccination	
	**Variables**	**OR (95%CI)**	**p-value**	**OR (95%CI)**	**p-value**	**OR (95%CI)**	**p-value**	**OR (95%CI)**	**p-value**	**OR (95%CI)**	**p-value**	**OR (95%CI)**	**p-value**	**OR (95%CI)**	**p-value**	**OR (95%CI)**	**p-value**
SD	Sex (Reference: female)			1.52 (0.88-2.64)	0.133					3.70 (0.84-16.40)	0.084			3.88 (0.70-21.38)	0.119		
	Practice in Western part of Germany (Reference: East)	2.81 (1.59-4.97)	**<0.001**	1.92 (0.89-4.14)	0.096	3.00 (1.37-6.55)	**0.006**	2.87 (1.48-5.55)	**0.002**								
	Amount of patients of at least 60 yrs < 50% (Reference: ≥50%)			2.15 (0.79-5.89)	0.143					2.19 (1.20-4.00)	**0.011**					4.05 (1.57-10.44)	**0.004**
	Amount of patients of ≥ 60 yrs (continuous variable)	0.99 (0.98-1.0)	0.117											0.97 (0.95-1.00)	0.023		
	Specialty: *No specialization*			0.21 (0.03-1.67)	0.14												
KL	Feels sufficiently informed regarding vaccinations for adults																
	Yes (Reference)			1 (ref)													
	Neutral			2.79 (1.16-6.69)	**0.022**												
	No			2.58 (0.51-13.15)	0.253												
Attitudes	Wants better information on changes of official recommendations																
	Yes (Reference)																
	Neutral									0.59 (0.25-1.40)	0.234					0.20 (0.04-1.05)	0.057
	No									0.86 (0.31-2.41)	0.772					0.34 (0.06-2.00)	0.234
	Trusts in official recommendations																
	Yes (Reference)	1 (ref)				1 (ref)		1 (ref)									
	Neutral	3.61 (1.56-8.32)	**0.003**			4.03 (1.54-10.57)	**0.005**	2.23 (0.93-5.34)	0.071	2.11 (0.84-5.29)	0.112			2.61 (0.63-10.83)	0.186		
	No	19.30 (2.28-163.24)	**0.007**			26.81 (2.96-243.12)	**0.003**	14.39 (2.87-72.31)	**0.001**	1.59 (0.20-12.74)	0.664			0.44 (0.02-12.71)	0.630		
	General objection of vaccinations																
	Yes (Reference)			1 (ref)													
	Neutral			0.70 (0.03-17.46)	0.83												
	No			0.16 (0.02-1.51)	0.11												
	Likes to counsel about vaccinations																
	Yes (Reference)	1 (ref)				1 (ref)		1 (ref)									

Neutral	2.94 (1.56-5.54)	**0.001**			2.20 (0.96-5.04)	0.062	2.38 (1.24-4.56)	**0.009**								
No	1.02 (0.24-4.27)	0.976			0.20 (0.01-5.05)	0.33	0.91 (0.21-3.97)	0.896								
Benefit of officially recommended vaccines exceeds its potential harms																
Yes (Reference)					1 (ref)		1 (ref)									
Neutral					0.80 (0.26-2.47)	0.703	0.39 (0.14-1.13)	0.083								
No					0.11 (0.02-0.65)	**0.015**	0.75 (0.24-2.31)	0.612								
Benefit of officially recommended influenza vaccine exceeds its potential harms																
Yes (Reference)					1 (ref)											
Neutral					3.82 (1.82-8.00)	**<0.001**							0.53 (0.12-2.29)	0.395		
No					17.81 (5.47-58.02)	**<0.001**							5.05 (1.45-17.57)	**0.011**		
Benefit of officially recommended pneumococcal vaccine exceeds its potential harms																
Yes (Reference)	1 (ref)						1 (ref)									
Neutral	1.85 (0.99-3.44)	**0.054**					1.95 (1.00-3.80)	**0.049**							1.25 (0.34-4.58)	0.734
No	3.26 (1.30-8.14)	**0.012**					2.81 (1.10-7.17)	**0.031**							23.74 (4.47-126.13)	**<0.001**
Regarding vaccinations, patients should be informed by:																
General Practitioner	0.31 (0.10-0.94)	**0.038**														
Medical specialist					1.57 (0.90-2.72)	0.11										
Health insurance company			3.09 (1.26-7.56)	**0.013**												
Physician Assistants			2.38 (1.20-4.74)	**0.013**												
Self-help/support groups for vaccine-preventable diseases															2.46 (0.70-8.70)	0.162
Media					0.66 (0.38-1.13)	0.129										
Public authorities									0.43 (0.21-0.90)	**0.026**						
Financial compensation for vaccinating and counseling is sufficient																
Yes (Reference)					1 (ref)											
Neutral					0.33 (0.15-0.73)	**0.006**										

	No					0.28 (0.14-0.54)	**<0.001**										
	There is not enough time to give council about vaccinations																
	Yes (Reference)																
	Neutral											0.14 (0.02-1.19)	0.072			0.57 (0.18-1.84)	0.351
	No											0.32 (0.09-1.12)	0.074			0.30 (0.10-0.93)	**0.037**
Practices	Not counseling routinely at regular intervals	2.86 (1.37-5.96)	**0.005**			3.10 (1.03-9.38)	**0.045**	5.32 (1.88-15.03)	**0.002**	2.19 (0.87-5.56)	0.098						
	Not counseling at first contact with patient															0.27 (0.09-0.79)	**0.017**
	Counseling due to injuries					3.22 (1.00-10.37)	0.05										
	Not counseling due to travel plans															21.46 (4.28-104.74)	**<0.001**
	Not counseling when indicated by recall-system			0.56 (0.32-0.99)	**0.047**							0.42 (0.13-1.29)	0.129			0.34 (0.13-0.89)	0.028
	Counseling is done by (reference: GPs and PAs)																
	only GPs			2.44 (1.20-4.96)	**0.014**												
	only PAs			Does not apply													
	Source of information regarding vaccines																
	Conferences														2.67 (0.86-8.28)	0.089	
	Continuous (online) training			1.75 (0.99-3.09)	0.055									3.53 (1.21-10.30)	**0.021**		
	Pharmaceutical representative									0.56 (0.30-1.03)	0.064	0.33 (0.10-1.03)	0.057		0.47 (0.19-1.15)	0.098	
	Professional journals			1.99 (1.01-3.94)	**0.048**	2.24 (1.15-4.37)	**0.018**	2.08 (1.20-3.59)	**0.009**			3.12 (0.96-10.15)	0.059			2.70 (1.03-7.09)	**0.043**
	Professional association											3.00 (0.67-13.36)	0.150			6.90 (2.00-23.84)	**0.002**
	Other source (not specified)					2.36 (1.11-5.05)	**0.026**			2.36 (0.96-5.77)	0.060						
	Does not seek information at all														16.77 (0.56-499.53)	0.103	

All variables with p<0.2 included in final models, statistical significant Odds Ratios (p<0.05) are shown in bold. SD=Sociodemographic and practice-characteristics, KL=Knowledge
